# Gait disorders are associated with non-cardiovascular falls in elderly people: a preliminary study

**DOI:** 10.1186/1471-2318-5-15

**Published:** 2005-12-01

**Authors:** Manuel Montero-Odasso, Marcelo Schapira, Gustavo Duque, Enrique R Soriano, Roberto Kaplan, Luis A Camera

**Affiliations:** 1 Division of Geriatric Medicine, McGill University, Montreal, Canada; 2 Geriatric Medicine Section, Hospital Italiano de Buenos Aires, Buenos Aires, Argentina; 3 Internal Medicine Department, Hospital Italiano de Buenos Aires, Buenos Aires, Argentina; 4 Informatics and Biostatistics Department, Hospital Italiano de Buenos Aires, Buenos Aires, Argentina; 5 Geriatric Fellowship, Faculty of Medicine, University of Buenos Aires, Buenos Aires, Argentina; 6 Solidage: McGill University, Université de Montréal Research Group on Integrated Services for Older Persons, Montréal, Canada

## Abstract

**Background:**

The association between unexplained falls and cardiovascular causes is increasingly recognized. Neurally mediated cardiovascular disorders and hypotensive syndromes are found in almost 20 percent of the patients with unexplained falls. However, the approach to these patients remains unclear. Gait assessment might be an interesting approach to these patients as clinical observations suggests that those with cardiovascular or hypotensive causes may not manifest obvious gait alterations. Our primary objective is to analyze the association between gait disorders and a non-cardiovascular cause of falls in patients with unexplained falls. A second objective is to test the sensitivity and specificity of a gait assessment approach for detecting non-cardiovascular causes when compared with intrinsic-extrinsic classification.

**Methods:**

Cross-sectional study performed in a falls clinic at a university hospital in 41 ambulatory elderly participants with unexplained falls. Neurally mediated cardiovascular conditions, neurological diseases, gait and balance problems were assessed. Gait disorder was defined as a gait velocity < 0.8 m/s or Tinetti Gait Score <9. An attributable etiology of the fall was determined in each participant. Comparisons between the gait assessment approach and the attributable etiology regarding a neurally mediated cardiovascular cause were performed. Fisher exact test was used to test the association hypothesis. Sensitivity and specificity of gait assessment approach and intrinsic-extrinsic classification to detect a non-cardiovascular mediated fall was calculated with 95% confidence intervals (CI95%).

**Results:**

A cardiovascular etiology (orthostatic and postprandial hypotension, vasovagal syndrome and carotid sinus hypersensitivity) was identified in 14% of participants (6/41). Of 35 patients with a gait disorder, 34 had a non-cardiovascular etiology of fall; whereas in 5 out of 6 patients without a gait disorder, a cardiovascular diagnosis was identified (p < 0.001). Sensitivity and specificity of the presence of gait disorder for identifying a non-cardiovascular mediated cause was 97.1% (CI95% = 85–99) and 83% (CI95% = 36–99), respectively.

**Conclusion:**

In community dwelling older persons with unexplained falls, gait disorders were associated with non-cardiovascular diagnosis of falls. Gait assessment was a useful approach for the detection of a non-cardiovascular mediated cause of falls, providing additional value to this assessment.

## Background

Falls are one of the giants of geriatric medicine, constituting a worldwide prevalent problem with substantial clinical and public health implications. Multiple risk factors have been identified as contributors to the fall syndrome and, accordingly, the list is highly heterogeneous including age-associated changes, neuro-sensory impairments, muscular weakness, comorbidities, cardiovascular mediated problems, polypharmacy, and environmental hazards, among others [1,2]. The most accepted classification of falls is based on whether risk factors are related to an intrinsic disorder or due to an extrinsic hazard [3,4]. However, a co-existence between these intrinsic and extrinsic factors has been found in almost 80 percent of elderly fallers limiting the clinical applicability of this classification [5]. When a principal intrinsic or extrinsic contributor is not clearly identified, patients with recurrent falls are classified as unexplained elderly fallers [2,6].

There is growing evidence of the association between unexplained falls and cardiovascular mediated causes [6-8]. Specifically, neurally mediated cardiovascular disorders and hypotensive syndromes account for up to 20 percent of these cases [8-11]. However, the approach to patients with unexplained falls remains unclear [2,12]. As clinical observation suggests that those with cardiovascular or hypotensive causes may not manifest obvious gait impairment, the assessment of gait impairments might be an interesting approach to these patients [3,10].

Accordingly, the principal objective of this study is to analyze the relationship between gait disorders and cardiovascular mediated falls under the hypothesis that gait disorders are mostly associated with a non-cardiovascular mediated falls. A second objective is to test the sensitivity and specificity of a gait assessment approach for detecting non-cardiovascular cause when compared with the intrinsic-extrinsic falls classification.

## Methods

### Study population and data collection

Consecutive patients referred to our Falls Clinic at "Hospital Italiano de Buenos Aires' between January 2001 and January 2002 were included in the study. All participants were community dwelling persons over 65 years of age with a history of recurrent and unexplained falls. A fall was defined as "an unintentional change in position resulting in coming to rest at a lower level or on the ground." [5]. Recurrent falls were defined as 2 or more falls in the previous six months [6]. Based on previous studies, unexplained falls were defined as falls without a principal cause attributed after a general practitioner evaluation [2,10]. A consent form was obtained for each participant.

Health and cognitive status, medications, and activities of daily living (ADLs) were recorded. Comorbidity was registered by a list of chronic diseases. A fall questionnaire, which included a list of intrinsic and extrinsic risk factors, place and circumstances of the fall, a list of comorbidities, use of psychopharmacological medication, level of physical activity and a history of fear of falling, was administered to each participant. A complete physical examination with supine and erect blood pressure measurement after 3 minutes of active standing, was also performed. All participants were assessed for neurally mediated cardiovascular conditions, neurological diseases, and gait and balance problems. In addition to a standard biochemical and hematological profile, a baseline electrocardiogram was performed in each participant. If diagnostic uncertainty remained, a head-up tilt test with carotid massage, a 24-hour holter electrocardiogram and 24-hour ambulatory blood pressure monitoring were performed. Further investigations, including echocardiography, knee or hip x-rays, computed tomography of the brain, were conducted as per published guidelines. [6] Two geriatricians, who had previously received training sessions and standardized instructions, conducted the assessments.

### Definitions and measurements at assessment

Based on previous studies, gait disorders were defined as either gait velocity (GV) equal or below 0.8 m/s or Tinetti Gait Test score below 9 [14-16]. Gait velocity was measured as the time taken to walk the middle 8 meters of 10 meters and was timed by a chronometer. The first and last meters, considered respectively warm-up and the deceleration phases, were not included in the calculation. Participants began the GV test on the word "go" and were instructed to "walk at a comfortable and secure pace." Each participant performed the task twice, with the final score being the time in seconds of the quicker of the two timed trials [14,15]. The Tinetti Gait Test is part of the performance-oriented assessment of mobility problems which assesses the following nine components: initiation of gait, step height and length, step symmetry and continuity, path deviation, trunk stability, walking stance, and turning while walking [17]. Each component was scored as 1 (normal) or 0 (abnormal) providing a final score which ranged from 0 to 12, with a higher score indicating a better gait performance.

Under the domain of neurally-mediated cardiovascular falls we included orthostatic and postprandial hypotension, vasovagal syndrome and carotid sinus hypersensitivity [10,11] Orthostatic hypotension was defined as a 20-mmHg fall in systolic, or 10-mmHg fall in diastolic BP during 3 minutes of active standing, whereas vasovagal syncope was defined when a head-up tilt test induced hypotension with or without bradycardia-asystole [10]. Carotid sinus hypersensitivity was defined as more than 3 seconds asystole (cardioinhibitory type), more than 50 mmHg fall in systolic BP (vasodepressor type), or both (mixed type) during carotid massage [9,10]. Postprandial hypotension was defined as hypotension (20-mmHg fall in systolic) within 2 hours of the start of a meal documented by 24 hour ambulatory blood pressure monitoring [10,11].

### Categorization after assessments

Each participant was classified as having an intrinsic or extrinsic fall using the information obtained at the fall assessment interview. Extrinsic falls were related to environmental hazards (slip, trip or externally induced displacement), whereas intrinsic falls were related to mobility or balance disorder, muscle weakness, orthopedic problems, loss of consciousness, neurally mediated cardiovascular disorder or sensory impairment [4]. According to the performance in gait velocity test and Tinetti Gait Test, each participant was classified as having a gait disorder or not. After a complete evaluation, an attributable etiology of the fall was determined. Following previous categorization, the attributable etiologies were grouped into four major categories including the neuromuscular, sensory, orthopedic and cardiovascular domains [7] (Table 1).

**Table 1:** Attributable etiology after fall assessment (n = 41)

**Domain**	**Principal cause**	**Number of patients**
**Neuromuscular**	Parkinsonism syndrome	3
	Unexplained Ataxia	2
	Lower extremity weakness	7
		
**Sensory**	Dizziness or vertigo	2
	Visual impairment	4
	Peripheral neuropathy	3
		
**Orthopedics**	Osteoarthritis (including foot problems)	8
	Hip problems or deformity	6
		
CV causes*	Orthostatic hypotension	2
	Postprandial hypotension	1
	Vasovagal syndrome	2
	Carotid sinus hypersensitivity	1

*CV causes: neurally mediated cardiovascular cause

### Statistical analysis

Associations between gait disorder and cardiovascular mediated diagnosis of falls were assessed using two-tailed fisher's exact test. A *p*-value ≤ 0.01 (two-sided) was considered statistically significant. Comparisons between intrinsic-extrinsic fall classification and gait assessment classification (presence or not of gait disorder) for diagnosing an attributable cardiovascular mediated cause were calculated with Chi-square test. Finally, sensitivity and specificity of each classification to detect a non-cardiovascular cause was evaluated. All calculations were performed with STATA 6.0 statistical package, Stata Corporation, College Station, Texas.

## Results

Forty-one consecutive patients with recurrent and unexplained falls were included. Average age was 79.63, SD ± 4 (range 70 to 95) and 73 percent were females. All subjects were ambulatory community dwelling older persons. Activities of daily living (ADLs) were normal for 85 percent of the subjects and mini mental status exam (MMSE) was normal in 78 percent (MMSE>26). The majority of the subjects could not describe a clear cause of their falls and all denied clinical characteristics compatible with syncope. Although six participants provided a clear description of the fall episode, it was insufficient for attributing an etiology to the fall. Average number of falls was 5 in the previous six months, with a range of 3 to 8. After a complete clinical and laboratory assessment, an etiological diagnosis was attributed to every participant as shown in Table 1. A neurally mediated cardiovascular disorder (orthostatic or postprandial hypotension, vasovagal syncope or carotid sinus hypersensitivity) was identified in 6 of the 41 patients (14%) as the main cause of their falls. Two patients were diagnosed with vasovagal syncope, two with postural hypotension, one with postprandial hypotension and one with carotid sinus hypersensitivity.

**Figure 1:**
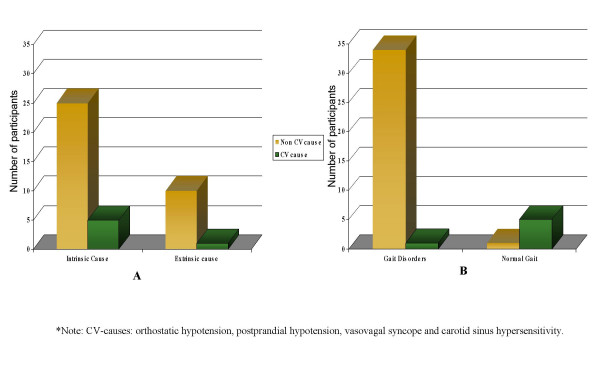
Distribution of fallers divided in cardiovascular mediated causes or not* according to extrinsic-intrinsic classification (A) or gait disorders presence (B).

Of the 35 patients with a gait disorder, 34 were diagnosed with a non-cardiovascular cause as the etiological diagnosis. By contrast, of the 6 patients without a gait disorder, 5 were diagnosed with a neurally mediated cardiovascular cause of fall (Table 2) (p < 0.001, fisher exact test). Distribution of the patients according to cardiovascular cause with both traditional and gait assessment classification is shown in Figure 1. Sensitivity and specificity of the presence of a gait disorder for diagnosing a non-cardiovascular cause was 97.1% (CI95% = 85–99) and 83% (CI95% = 36–99), respectively. Sensitivity and specificity of intrinsic-extrinsic classification for diagnosing a non-cardiovascular mediated cause was 28% (CI95% = 14–46) and 83% (CI95% = 36–99) respectively (Table 2).

**Table 2:** Diagnostic accuracy of gait disorder and intrinsic-extrinsic classification for detecting non-cardiovascular mediated falls (n = 41)

**Diagnostic Approach**	Patients with Non-CV Causes*	**Patients with CV Causes**†	**Sensitivity (%)**	**Specificity (%)**	***p*-value** Fisher exact test
Gait Disorder +	34	1	97.1	83	<0.001‡
Gait Disorder -	1	5			

Extrinsic Fall	10	1	28	83	0.9128‡
Intrinsic Fall	25	5			

*Non-CV cause: non-cardiovascular neurally mediated cause. †CV causes: cardiovascular neurally mediated cause. Gait Disorder +: presence of gait disorder. Gait Disorder -: absence of gait disorder. ‡p-value of the associations with non-cardiovascular mediated falls for each diagnostic approach.

Note: Neurally mediated cardiovascular causes are the following: orthostatic hypotension, postprandial hypotension, vasovagal syncope and carotid sinus hypersensitivity.

## Discussion

In this sample of ambulatory patients referred with unexplained falls, the presence of a gait disorder was significantly associated with a non-cardiovascular cause of falls. Of the 35 participants with a gait disorder, 34 had a non-cardiovascular mediated fall. By contrast, 5 out of 6 participants with a cardiovascular mediated cause had a normal gait performance. This interesting observation suggests that when gait disorders are not detected in patients with unexplained falls, a cardiovascular mediated origin should be seriously considered.

Although intrinsic-extrinsic categorization was intended to separate and identify multiple contributors to the fall, this classification provides limited clinical help since older people who experience an extrinsic fall often have an underlying intrinsic condition that decreases their ability to compensate for the hazardous situation [7]. Rather, falls are often related to a complex interaction among intrinsic and extrinsic factors which challenges the postural control and the ability to maintain an upright position. In our study, the intrinsic-extrinsic categorization did not provide additional information for detecting a cardiovascular mediated cause. In fact, when these 41 participants with unexplained falls were analyzed under this categorization, we found a similar pattern of distribution regarding cardiovascular cause with a high non-cardiovascular/cardiovascular cause ratio in both groups (Figure 1A). In contrast, when using the gait assessment approach, the distribution showed almost no gait disorder when falls were associated with a cardiovascular origin with an inverse ratio. (Fig 1B). For instance, 5 out of 6 patients with the attributable diagnosis of a cardiovascular cause had a normal performance in the Tinneti Gait Test and a gait velocity above 0.8 m/s.

Why should gait assessment be considered a key component in the assessment of unexplained falls? Gait performance is a fascinating and complex task that depends on the normal functioning of multiple systems working in a highly coordinated and integrated manner [14,16]. As impairments in different domains can alter this delicate system, it is interesting to hypothesize that different chronic conditions such as visual or hearing problems, muscular weakness, osteoarthritis or peripheral neuropathy could be evidenced through gait performance. Moreover, the use of benzodiazepines, neuroleptics, and other drugs with central action may affect gait performance. Chronic cardiovascular diseases and systemic chronic hypertension have been also associated with continuous impairment in gait performance [18,19]. However, falls secondary to neurally-mediated cardiovascular causes may be expressed by a different mechanism, without necessarily chronically affecting gait performance [10,15]. Regulation of systemic blood pressure is important for postural control in elderly people since failure to perfuse the brain increases the risk of falls. Additionally, age-related decline in baroreflex sensitivity contributes to the vulnerability in changing posture or in the postprandial state [20,21]. Although the exact mechanism by which a neurally mediated cardiovascular problem causes a fall remains unclear, there is growing clinical evidence for its association with unexplained falls [22].

Interestingly, participants in our study who were diagnosed with a cardiovascular cause of falls denied the clinical symptoms traditionally associated with a syncopal phenomenon. This observation agrees with the increasingly detected overlap between syncope and falls in the subgroup of unexplained elderly fallers [9]. A possible explanation may be the incapacity to remember syncopal symptoms due to a retrograde amnesia of the episode [9,10,13].

Since a cardiovascular mediated cause of fall has been described in up to 20 percent of unexplained elderly fallers, and in view of the associated morbidity, mortality and the availability of treatment, careful consideration should be given when assessing patients with unexplained falls [9,10]. As well, falls related to a neurally mediated cardiovascular event could be expressed acutely and intermittently without chronically affecting gait performance, providing a potential explanation for the absence of gait disorders in these participants. As gait performance can be assessed directly, thus avoiding reporting bias, this approach could be applied even in absence of a detailed description of the episode. Finally, the simplicity of the proposed gait assessment makes it easy to perform and accessible for general clinicians and other health professionals.

Our study has the strength of a systematic and comprehensive assessment of the participants who were diagnosed with a potential etiology of falls; however, important limitations should be outlined. The sample studied comprised a select and relatively small number of participants with a low proportion of them with cardiovascular mediated falls, facts which affect the generalizability of the findings. Due to the design characteristics, causality was not pursued and only an association between the cardiovascular diagnosis and the fall episode can be described. A prospective and controlled trial in a larger sample is needed to strengthen our hypothesis.

## Conclusion

In ambulatory older persons with unexplained falls, the presence of a gait disorder was associated with non-cardiovascular mediated falls. Gait assessment was useful for the detection of non-cardiovascular causes of falls, providing an additional diagnostic yield to this assessment. This clinical based approach may help on the evaluation of elderly persons with unexplained falls.

## Authors' contributions

MMO and MS collected the clinical data. All six authors participated in the design of the study. MMO and ERS did the analyses. MMO, ERS and GD wrote the first draft of the manuscript. All of the authors reviewed the analyses, and read and approved the final manuscript. The authors declare that they have no competing interests.

## Pre-publication history

The pre-publication history for this paper can be accessed here:


